# Pathogens as commensals: microbial priming of the immune system and heterologous protection

**DOI:** 10.1099/mic.0.001680

**Published:** 2026-02-26

**Authors:** Thomas Belcher, Emily J. Stevens

**Affiliations:** 1 School of Life Sciences, Keele University, Keele, UK

**Keywords:** bacterial pathogenesis, commensalism, microbiome, opportunistic pathogens

## Abstract

Exposure to microbes is essential to promote the development of the host’s immune system. Commensal microbes (i.e. the microbiota) which are acquired early in life play a vital role in immune priming. Whilst many organisms within the microbiota are harmless, some can be considered opportunistic pathogens. Examples include *Staphylococcus aureus*, *Streptococcus pneumoniae* and *Pseudomonas aeruginosa*, and these organisms can also contribute to the development of a healthy host immune system. At the extreme end of the spectrum, pathogens which typically do not form part of the microbiota (e.g. *Mycobacterium tuberculosis*, *Bordetella pertussis* and *Salmonella* Typhimurium) have been shown to provide cross-protection against infectious and non-infectious diseases in mice. Attenuated strains of these pathogens, such as BPZE1, could have clinical applications, whilst Bacillus Calmette–Guérin, a live-attenuated *Mycobacterium bovis* strain, has been shown to have non-specific effects against cancers and other diseases. A wide range of organisms, from harmless microbiota to potentially life-threatening infections, interact with the host immune system and can prime or modulate the immune response in different ways. In this review, we discuss the important role that pathogens, including opportunistic components of the microbiota, play in the development and maintenance of host immunity to a wide range of infectious and non-infectious diseases.

## Introduction

Microbes, acquired from the beginning of life, are vital to the development of a healthy host immune system [1,3]. Commensal microbes (those that colonize a host without being either harmful or overtly beneficial) have been shown to aid development of the innate and adaptive immune system, including priming of immune cells and stimulation of antibody production [3,4]. They also contribute to the maintenance of the gut epithelial barrier [4] and themselves provide a physical barrier to infection in the form of colonization resistance [5]. Exposure to pathogens also contributes to the development of immune functions throughout life [6,8].

It is possible to simulate beneficial immune responses to infectious agents and to generate specific immune memory to pathogens through vaccination. Vaccination has proven to be highly effective in mimicking microbial priming of the immune system through antigen exposure without the need for infection with the pathogen. Live-attenuated vaccines demonstrate an ability to promote the development of host immunity and in some cases provide protection against more than their initial intended target, referred to as heterologous protection, off-target effects or non-specific effects. For example, the live-attenuated measles vaccine reduces all-cause child mortality (up to 5 years of age) by 30–86% across a number of studies, which is more than the proportion of deaths that can be attributed to acute measles disease [9]. These heterologous effects of live-attenuated vaccines are comparable to heterologous protection caused by infection with some pathogenic microbes. This tallies with the central dogma of the hygiene/‘old friends’ hypothesis, which postulates that an overly sanitized, primarily indoor upbringing with reduced exposure to microbes precipitates the onset of allergies and autoimmune diseases [10,11].

Exposure to pathogens, whether through live-attenuated vaccination or natural infection, can thus be beneficial in the context of the developing immune system. However, where infections are known to confer significant morbidity and may have a high mortality rate, the trade-off between benefits to immune development and risk to life or quality of life is highly imbalanced. As alternatives, organisms considered to be ‘opportunistic pathogens’ – those that live commensally within the host microbiota but can take advantage of changes in host health to cause infection – might have been overlooked as valuable contributors to the development and maintenance of a healthy immune system. A better understanding of the mechanisms by which such organisms may benefit their host in the commensal state will help to understand why these organisms are maintained within the microbiota and to identify the factors that contribute to their evolvement as invasive pathogens in some contexts. Likewise, a better understanding of how pathogens and parasites more broadly generate beneficial immune responses, whether to the infecting organism itself or to heterologous pathogens and various non-infectious diseases, will enable the development of better prevention and treatment strategies.

As microbes are increasingly being explored as preventative and therapeutic agents to tackle infectious and non-infectious diseases, attenuated pathogens may provide an important avenue of investigation in generating broad, beneficial priming of the host immune system. Here, we discuss a range of examples of opportunistic pathogens, pathogens and parasites without a commensal aspect to their lifecycle and attenuated pathogens, focusing on how they confer benefits to the development of host immunity. We use the terms ‘pathogen’ and ‘parasite’ interchangeably for the purpose of this review. We highlight the current knowledge gaps relating to the mechanisms by which these organisms benefit the host and discuss the potential for such organisms to be further adapted to develop improved prevention and treatment strategies for infectious and non-infectious diseases.

## Opportunistic pathogens, as commensal components of a healthy microbiota, benefit their host through immune priming

It is a well-established paradigm that infection by pathogens, if survived, in most cases provides some degree of immunity to future infection by the same pathogen. Opportunistic pathogens are no exception. In a study from 1965, it was reported that a ‘relatively non-pathogenic’ *Staphylococcus aureus* strain (502A) was used to control an outbreak of highly virulent *S. aureus* in a neonatal nursery [12]. This procedure was described as ‘bacterial interference’, in which colonization by strain 502A prevented the later acquisition by infants of other, more virulent, staphylococcal strains.

A later clinical study [13] examined the effect of *S. aureus* carriage on the outcome of hospital-acquired *S. aureus* bacteraemia. In this study, non-surgical hospital patients were screened for nasal *S. aureus* carriage and monitored for the onset of *S. aureus* bacteraemia. Whilst patients who carried *S. aureus* in their nose had a higher risk of developing bacteraemia, non-carriers were found to have a higher risk of mortality from *S. aureus* bacteraemia than carriers. Underlying differences in comorbidities or age were ruled out as causes for the difference in mortality risk. These findings suggest that carriers of *S. aureus* in the nose are more immunologically adapted to *S. aureus* than non-carriers. As ~80% of *S. aureus* bacteraemia infections are of endogenous origin [13,15], carriers appear thus to benefit from immune priming by their commensal *S. aureus* strain, decreasing risk of death from *S. aureus* bacteraemia caused by the carriage strain, whilst non-carriers must mount a fresh immune response to an unfamiliar invading pathogen. Alternatively, the potential higher virulence of exogenous *S. aureus* strains may explain the increased mortality risk in non-carriers upon onset of *S. aureus* bacteraemia.

Similar to *S. aureus* [13], nasopharyngeal colonization by *Streptococcus pneumoniae* has been demonstrated to protect against subsequent lethal pneumonia through priming of the host immune system [16]. In mice, nasopharyngeal colonization with *S. pneumoniae* primed a range of immune responses, including Th17-polarized cytokine production and the generation of antibodies, found to protect against subsequent infection. In this case, protection was provided against subsequent infection with the same strain of *S. pneumoniae*. However, cross-protection against heterologous *S. pneumoniae* strains as a result of prior infection has also been demonstrated and was mediated by memory Th17-polarized T-cells [17]. In contrast, immunization with inactivated *S. pneumoniae* provided less effective protection against heterologous *S. pneumoniae* strains [18]. Thus, active colonization with *S. pneumoniae* appears to confer more robust immunological priming. Carriage rates of *S. pneumoniae* in young children are high (ranging from 26–90% in different parts of the world [19,21], and *S. pneumoniae* is responsible for the third highest burden of mortality from bacterial disease globally [22]. However, rates of pneumococcal septicaemia decrease rapidly in older age groups, potentially as a direct result of the protective effects of initial colonization, or repeated colonization by various serotypes in early life [16].

Similar effects of immune priming against disease of endogenous [13] or exogenous origin by the same species [16] have been observed across a range of opportunistic pathogens [23,24]. An increasing number of studies have also identified the potential for pathogens to confer heterologous protection against unrelated pathogen species. This has the potential to open new avenues of investigation to identify strategies to protect against, or treat, infectious and even non-infectious disease.

### *S. aureus* can provide protection against infectious disease

Many medically important pathogens live mostly as commensals within a host. *S. aureus* is a well-studied opportunistic pathogen known to cause a variety of infectious diseases, ranging from minor skin and soft tissue infections to severe infections of the respiratory tract, heart and bloodstream [25,26]. Justifiably, the focus of *S. aureus* research has primarily been on its pathogenic capabilities. However, it is also well known that *S. aureus* predominantly colonizes the human host as an asymptomatic commensal of the nose, skin and/or gut [27,30]. In this capacity, it is effectively a member of the microbiota. Its role in this setting is less well defined [31,33], but it is possible that *S. aureus* fulfils a role similar to that of other beneficial microbes [34].

Peres Emidio *et al*. [35] recently demonstrated that *S. aureus* infection protects against subsequent co-infection with the fungus *Cryptococcus gattii* in mice. Stimulation of an inflammatory response by prior intranasal *S. aureus* infection was found to promote clearance of *C. gattii* upon subsequent co-infection. Priming by *S. aureus* accelerated the expression of proinflammatory cytokines and the recruitment of macrophages and neutrophils upon secondary infection, compared to *C. gattii* infection alone. *S. aureus* did not directly inhibit *C. gattii* growth but elicited a broad non-specific protective immune response in the immediate aftermath of initial *S. aureus* infection.

Immune priming by *S. aureus* (and other pathogens, see ‘Pathogens and parasites protect against infectious diseases’ section) also protected against influenza infection in the respiratory tract [36]. In mice, priming with *S. aureus* led to recruitment of peripheral blood CD11b^+^ monocytes involved in the immune response to influenza, which were maintained after the initial priming and protected mice against subsequent influenza-mediated lung injury and death [36]. The authors highlighted epidemiological studies showing that the 2009 H1N1 (‘swine flu’) pandemic caused higher morbidity and mortality in high-income countries, compared to low- and middle-income countries [37,38], supporting the hygiene hypothesis [10]. A reduction in microbiome diversity in people living in high-income countries, due to a sanitised lifestyle, may contribute to increased susceptibility to both infectious and non-infectious diseases [39,41].

### Examples of heterologous protection from other opportunistic pathogens against infectious disease

Heterologous protection against subsequent infection with bacterial pathogens has also been demonstrated by the opportunistic pathogen *Candida albicans*. In a mouse model of candidiasis, infection by a *C. albicans* mutant deficient in the transcription factor Flo8p, required for development of hyphae, protected not only against subsequent lethal *C. albicans* infection but also against broader polymicrobial sepsis [42]. The immune mechanism was Th1-dependent, since administration of anti-CD4 or anti-IFN-γ antibodies blocked the protective effects.

**Fig. 1.**
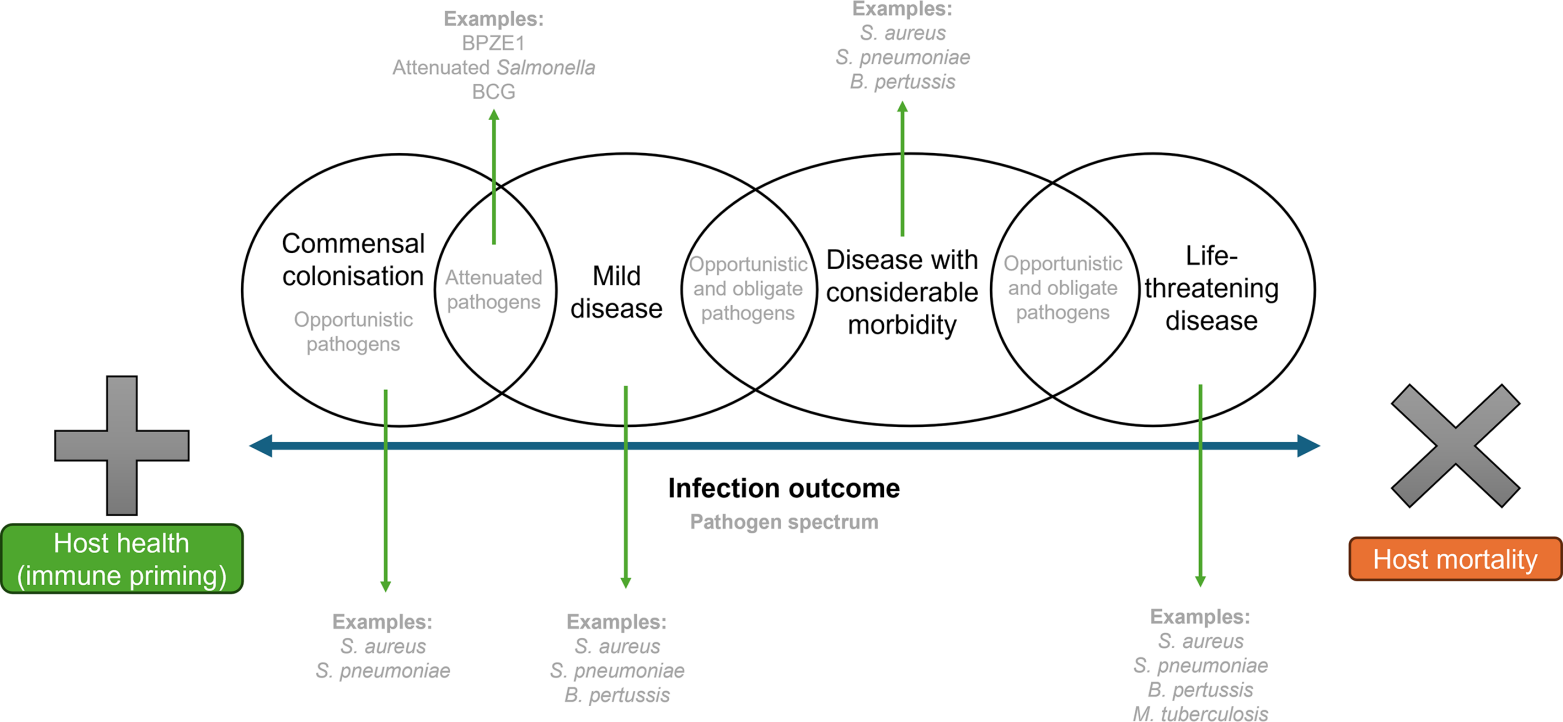
The spectrum of infection outcome, which spans from beneficial immune priming (to which both opportunistic and attenuated pathogens can contribute and to some degree obligate pathogens depending on the severity of infection) to host mortality.

The capacity for microbes to transition between pathogenic and ‘beneficial’ has been evidenced through an *in vivo* evolution experiment in nematodes [43]. In this model, following serial passage through successive host generations, a mildly pathogenic *Enterococcus faecalis* strain transitioned to a state of mutualism and protected the worm against *S. aureus* infection. This supports the concept that in particular contexts, microbes considered to be ‘pathogens’ can transition to commensalism and confer heterologous protection against other pathogens. The reverse can also occur, as shown in a recent review highlighting how, in certain contexts, the traditionally beneficial microbiota can facilitate pathogen infection [44]. Context can therefore be considered the defining factor in determining the outcome of microbe–host interactions, making such outcomes difficult to predict (Fig. 1) and in turn manipulate. Efforts to develop protective microbes for preventative and therapeutic use in the treatment of disease will need to consider how the context of host–microbe interactions can affect intervention outcomes.

### *S. aureus* can provide protection against non-infectious disease

In addition to providing heterologous protection against infectious disease, *S. aureus* has been found to provide protection against allergic rhinitis [45]. Inoculation of *S. aureus* derived from patients with allergic rhinitis was found to downregulate Th2 cytokines and IL-33 expression in both an epithelial cell model and a mouse model [45]. In mice, this resulted in a reduction of allergic symptoms, and the authors go so far as to discuss the possibility of using *S. aureus* itself in a probiotic capacity to treat allergic rhinitis. A seemingly contradictory finding is that patients with allergic rhinitis have higher levels of colonization by staphylococcal species (including *S. aureus* and *Staphylococcus epidermidis*) than non-allergic subjects. This apparent contradiction may be because allergic inflammation benefits *S. aureus* (and other staphylococcal commensals) by creating an environment in which it can thrive, although *S. aureus* in patients with allergic rhinitis may itself be protective against allergic inflammation. The timing of *S. aureus* colonization may be the determining factor in whether *S. aureus* prevents allergic rhinitis onset or colonizes an already inflamed host environment. Similarly, prevention of asthma by Bacillus Calmette–Guérin (BCG) may depend on how early in life one is vaccinated, as studies suggest that BCG is more effective at preventing allergic disease the earlier the vaccine is administered [46].

The examples provided here throughout the ‘Opportunistic pathogens, as commensal components of a healthy microbiota, benefit their host through immune priming’ section illustrate how cross-talk between an opportunistic pathogen and the host can prime the immune system, providing heterologous protection against infectious and non-infectious diseases [47]. In the case of *S. aureus,* disease may be less severe as a direct result of prior *S. aureus* carriage. However, as carriage of *S. aureus* comes with an inherent risk of endogenous disease [13,15], management of *S. aureus* colonization is a complex issue. Efforts to eliminate *S. aureus* from the nasopharynx and other niches, whilst important for patients who might be vulnerable to disease, might be detrimental to an otherwise healthy host. Further research into the role of *S. aureus* as a commensal colonizer may yet identify other benefits of its carriage beyond immune priming and provide insights into the mechanism of its persistence in the nasal microbiota in a subset of the population [33,34] (see inset Box 1).

Box 1. **Maintenance of opportunistic pathogens within the microbiota, using S. aureus as an example: implications for health and infection prevention**

A healthy microbiome consists of a diverse mix of so-called ‘beneficial’, ‘commensal’ and ‘opportunistic’ microbes, along with viruses, archaea and eukaryotes [108,109]. As such, studying opportunistic pathogens solely from the perspective of pathogenesis is to miss a large part of their lifestyle. Microbial transitions to pathogenicity in patients that become endogenously infected start from a state of commensalism, surrounded by numerous other microbes. It is in this context that there is room for new knowledge, especially on the mechanisms underlying opportunism and the various benefits for their hosts.

Whilst opportunistic pathogens may contribute directly to priming of host immunity, they also provide resilience against infection through promoting a healthy microbiota, for example by providing colonization resistance against invading pathogens [12]. A better understanding of how these opportunists contribute to the host microbiota in their commensal form may help to elucidate the factors that determine their colonization. In turn, this may aid evaluation of how best to manage colonization in vulnerable patients (for example, decolonization of S. aureus in patients awaiting surgery), which may reduce reliance on current antibiotic and antiseptic methods [30].

Characterization of healthy microbiota communities is an active area of research. Liu et al. [110] identified seven community state types (CSTs) among the nasal microbiomes of healthy human participants. Each CST was defined by the dominance of particular bacterial species, and for CST1, the dominant species was the opportunistic pathogen S. aureus. This finding is also supported by data from more recent research [111]. Furthermore, across different CSTs, the presence of S. aureus can be predicted by the presence or absence of specific taxa. For example, Propionibacterium granulosum and Corynebacterium spp. were negatively correlated with the presence of S. aureus, whilst S. epidermidis was positively correlated with S. aureus. Dolosigranulum spp. predicted S. aureus presence in a threshold-dependent manner. Host genetics was found to have a limited impact on microbiota community composition. These data illustrate that not only is S. aureus colonization linked to wider microbial community dynamics, but that in some people, it is a dominant component of the healthy microbiota (i.e. in CST1). Where this is the case, it could be argued that S. aureus confers a benefit on the host and/or wider microbiota which might select for its enrichment, and in turn dominance, in the community.

Several other studies have also characterized microbiota community dynamics in relation to S. aureus carriage. Across the literature, various bacterial species have been linked to promoting or restricting S. aureus colonization. Dolosigranulum pigrum, for instance, has been shown to have antimicrobial activity against S. aureus [112]. Corynebacterium accolens has been found to compete against S. aureus, reducing its capacity to bind to epithelial cells [113], having antimicrobial activity against S. aureus [113,114] and inhibiting mucosal barrier disruption induced by S. aureus [115]. More broadly, Corynebacterium spp. have also been shown to stimulate a shift towards commensalism in S. aureus [116]. Bacillus spp. have been shown to inhibit S. aureus biofilm formation and proliferation in animal hosts [117], and more recently to exclude S. aureus from the intestines of animal and human hosts through quorum-sensing interference [118,119]. Propionibacterium acnes, on the other hand, has been found to promote staphylococcal aggregation and biofilm formation [120,121].

The current S. aureus CARRIAGE study [111] has characterized the nasal microbiome of 1,180 participants, to determine the microbiome structure associated with S. aureus carriage. Their findings demonstrate, in line with the findings of Liu et al. [110], a distinct CST, dominated and shaped by S. aureus, and an additional six CSTs in which S. aureus is rare or absent. The authors further demonstrate an ability to predict S. aureus colonization status from their data using machine learning, with high sensitivity in predicting non-carriage. This will be particularly useful in evaluating the risk of persistent colonization in patient groups and may ultimately facilitate targeted decolonization approaches to limit the use of antibiotic-based methods [122,123].

In their review of the commensal lifestyle of S. aureus, Krismer et al. [31] highlight that commensalism in this and other opportunistic bacteria has been a neglected area of research. Whilst it is important to study the factors that drive pathogenesis in opportunists like S. aureus, understanding the dynamics of S. aureus carriage is becoming equally valuable. In this way, it may be possible to better understand why some people are predisposed to carry S. aureus (the work of Aggarwal et al. in the CARRIAGE study suggests the microbiota is a major determinant), and in turn, this may aid the identification of risk factors for endogenous disease.

## Pathogens and parasites protect against infectious and non-infectious diseases

### Pathogens and parasites protect against infectious diseases

In mice, infection with the intestinal parasite *Giardia intestinalis* protected against disease caused by infection with the unrelated parasite *Toxoplasma gondii* administered 3 days later [48]. *Giardia*-infected mice exhibited a reduction in IFN-*γ* production and an increase in IL-10 production. In the same study, *Giardia* infection also protected against dextran sulphate sodium-induced acute colitis, reducing mucosal ulceration and inflammatory infiltrates. Protection in both models was likely due to the robust stimulation of Th2 cells secreting IL-10, which promoted *Giardia* replication and persistence but was protective against gut inflammation, suggesting that the parasite induces a tolerogenic environment in which to thrive. In humans, there is some evidence linking *Giardia* infection with reduced risk of severe diarrhoea from other pathogens [49].

Soil-transmitted helminths have been associated with reduced severity of COVID-19 disease in humans through potential immune priming [50]. Soil-transmitted helminths, including ascarids, whipworm and hookworm, trigger a Th2 response, resulting in immunosuppression, decreased expression of the ACE2 receptor targeted by SARS-CoV-2 and reduced inflammatory Th1/Th17 responses [50]. COVID-19 vaccines were designed to promote a Th2 response, such that the hyperinflammatory Th17 response was countered, reducing immunopathology from COVID-19 disease [51]. In the same manner, soil-transmitted helminths may reduce the severity of COVID-19. This is supported by evidence indicating that co-infection with helminth parasites reduces the risk of severe COVID-19 [52], with reduced rates of COVID-19 mortality in geographic regions where helminth infection is prevalent [53].

Infection with the respiratory pathogen *Bordetella pertussis* has been shown to exacerbate subsequent influenza infection in mice [54]. This finding was specifically linked to pertussis toxin (PT). Mice that were infected with *B. pertussis* followed by influenza up to 14 days later had increased viral titres, lung pathology and mortality compared to mice that received a PT-deficient strain of *B. pertussis* followed by infection with influenza. In contrast, a different study showed that mice infected with *B. pertussis* were protected against influenza-induced disease and death [55]. Curiously, *B. pertussis* strains expressing enzymatically active PT significantly reduced the viral load of subsequent influenza virus infection, an effect that was correlated with an increase in the levels of IL-17A in murine lungs. The key difference between the studies appears to be the timing of influenza infection following *B. pertussis* administration. In the former study [54], the virus was administered up to 14 days after *B. pertussis*, whereas in the latter [55] the interval was 21 days, which is beyond the peak of *B. pertussis* infection in the mouse, suggesting that once *B. pertussis* infection is resolved, there exists a state of protection against subsequent influenza disease and infection.

### Pathogens and parasites protect against non-infectious diseases

Parasite infection has been shown to promote non-specific immune priming in mice and humans. Exposure to eggs of the parasite *Schistosoma mansoni* prevented type 1 diabetes (T1D) in mice [56]. Four injections of dead *S. mansoni* eggs 1 week apart were sufficient to prevent T1D in non-obese diabetic (NOD) mice but only if the injection course was started when the mice were 4 weeks old, before the onset of pancreatic infiltration. This contrasts with the protection against diabetes provided by attenuated *Salmonella* strains discussed in the ‘Attenuated pathogens protect against non-infectious diseases’ section, which was observed until the mice were at least 12 weeks old. Additionally, *Salmonella*-mediated protection was only achieved with live bacteria, as heat-killed *Salmonella* did not provide protection against diabetes [57]. Soluble schistosome extracts were shown to have the same effect as killed eggs, and a role for IL-10 was suggested.

## Attenuated pathogens protect against infectious and non-infectious diseases

### Attenuated pathogens protect against infectious diseases

The finding that virulent pertussis can protect against influenza disease in mice (discussed above) [55] followed a number of studies investigating BPZE1, a novel live-attenuated pertussis vaccine candidate that is now in late-stage clinical development [58,62]. The vaccine is given intranasally and is a genetically modified mutant of *B. pertussis* deficient for tracheal cytotoxin and dermonecrotic toxin and producing genetically inactive (PT [58]. Early work investigating the potential of BPZE1 to protect against the related species *Bordetella bronchiseptica* in mice showed a cross-species protective effect, which was mediated in part by the induction of CD4^+^CD25^+^FoxP3^+^ regulatory T-cells in addition to cross-reactive CD4+ effector T-cells [63]. Additionally, it was shown that protection by BPZE1 against virulent *B. pertussis* could be observed within days of vaccination, before antibody or specific T-cell responses were detectable [64]. This was due to TLR4-dependent signalling through the MyD88 pathway.

BPZE1 was also shown to protect against influenza disease when the virus was administered 6 weeks after BPZE1 [65], however, without decreasing viral load. Instead, BPZE1 prevented the virus-induced cytokine storm and protected against influenza pathology in the lungs, against weight loss and mortality. Protection could be boosted by a second dose of BPZE1 which enhanced the protection against influenza. The protective effect was shown to last for at least 12 weeks post-BPZE1 administration, long after the bacteria had cleared in mouse tissues. Sera from pertussis-infected mice did not cross-react with influenza virus, and splenocytes from BPZE1-immunized mice did not proliferate or produce IFN-*γ* upon stimulation with viral particles, suggesting that the mechanism underlying BPZE1-mediated protection against influenza did not depend on adaptive immunity.

BPZE1 also protected mice from a challenge with respiratory syncytial virus (RSV) [66]. Priming with BPZE1 14 days before RSV challenge led to the abolition of weight loss and a reduction of RSV copy numbers in the lung. The reduction in pathogen load is in contrast to the lack of effect of BPZE1 on influenza viral load but recalls the effect of virulent *B. pertussis* on influenza infection in mice [55]. No antibody or T-cell cross-reactivity was detected between *B. pertussis* and RSV, again suggesting that the non-specific effect of BPZE1 was independent of adaptive immunity. BPZE1 reduced the influx of immune cells in murine lungs following RSV infection but increased the numbers of CD4^+^ T-cells expressing IL-17, which was shown to be necessary for the protective effect. The effect was long-lasting, since priming neonatal mice (2–5 days old) with BPZE1 protected them against RSV challenge into adulthood. Similar to the protection by attenuated *B. pertussis* against RSV challenge, virulent *B. pertussis* infection has been associated with reduced RSV disease severity in young children [67].

Additionally, it was shown that two doses of BPZE1 (but not one dose) protect against challenge with *S. pneumoniae* [68]. Mice were protected against pneumococcal colonization of the lungs, disseminated infection in the blood and spleen, and against weight loss and mortality. In this model, two doses were necessary to observe protection, suggesting a prime–boost effect. In this prime–boost regimen, priming had to occur with live BPZE1 to induce protection against pneumococcal pneumonia, whereas the second dose could be administered as live or heat-killed bacteria. In contrast to the BPZE1-mediated protection against influenza and RSV, protection against pneumococcal pneumonia was short-lived, waning after 3 days post-immunization with BPZE1.

The mechanisms of BPZE1-mediated protection against heterologous infectious diseases are not clear. In some models, heterologous protection was accompanied by a drop in heterologous pathogen load, such as for *S. pneumoniae* and RSV, and in some models, this was not the case, such as for influenza. It would therefore appear that BPZE1 induces distinct mechanisms of heterologous protection, most likely all involving the innate immune system. In the case of invasive pneumococcal disease, the BPZE1-mediated effects depended on MyD88 signalling [68]. The mechanism of this short-lived protective effect may be similar to that of intranasal administration of bacterial flagellin, which also protects against subsequent lethal infection with *S. pneumoniae* for up to 24 h [69]. Flagellin induces the expression of pro-inflammatory genes and subsequent neutrophil infiltration. It is tempting to assume a similar mechanism in the case of BPZE1. However, *B. pertussis* does not express flagellin due to frame-shift mutations in its flagellin gene, although one study has shown that it can express flagellum-like structures under certain circumstances *in vitro* [70]. Alternatively, other innate-immune stimulators, such as lipid A, may induce a similar pro-inflammatory response in the lungs and play a part in resistance to *S. pneumoniae*. In the case of BPZE1-mediated protection against influenza, in which protection is longer lasting and is not accompanied by a drop in heterologous pathogen load, protection would depend more on a tolerogenic effect, in which the typical immunopathology accompanied by the cytokine storm typical of severe influenza infection is dampened. The exact immune mechanisms involved remain to be elucidated. A summary of studies investigating non-specific protection by BPZE1 against a range of infectious and non-infectious diseases is presented in Table 1.

**Table 1.** A summary of studies related to the non-specific effects of the live-attenuated pertussis vaccine candidate BPZE1 in mouse models of infectious and non-infectious disease

	Model	Does BPZE1 reduce pathogen load?	Was a booster effect observed?	Was the effect long-lasting?	Potential mechanism	Did virulent pertussis protect in this model?	Reference(s)
Non-specific protection by BPZE1 in models of infection	*B. bronchiseptica*	Yes	Not reported	At least 4 weeks	Cell-mediated (Th1/Th17 T-cells), dependent on CD4+CD25+FoxP3+ regulatory T-cells, possible role of antibody (some cross protection between *B. bronchiseptica* and *B. pertussis*	Not reported	[63]
	Influenza	No	Yes	At least 12 weeks	Unknown, but protection by virulent pertussis is through a distinct mechanism which is IL-17- and PT-dependent	Yes, though a distinct IL-17-dependent mechanism associated with a reduced pathogen load	[55,65]
	RSV	Yes	Not reported	Yes, neonates are protected into adulthood	IL-17-dependent, possible enhanced maturation of dendritic cells	Not reported	
	Pneumococcal pneumonia	Yes	Yes	No, wanes after 3 days	Unknown, but MyD88-dependent	Not reported	[68]
	Model	Effect of BPZE1	Was a booster effect observed?	Was the effect long-lasting?	Potential mechanism	Did virulent pertussis protect in this model?	Reference(s)
Non-specific protection by BPZE1 in models of non-infectious disease	OVA-induced asthma	Reduction in total cell, neutrophil, eosinophil and lymphocyte recruitment and Th2 cytokines (IL-5, IL-13 and IL-4) in bronchiolar lavage	Not reported	At least 6 weeks	Unknown	No	[74,75]
	Contact dermatitis	Reduction in ear thickness and inflammatory cytokines (IL-1*β*, IL-6, IL-2, TNF-*α*, IL-17, IL-4) in ear homogenates	Yes	At least 6 weeks	Unknown	Not reported	[75]
	HDM-induced allergic airway inflammation	Reduction in airways resistance; eosinophil recruitment and serum HDM-specific IgG1	Yes	At least 4 weeks	Unknown, although distinct mechanisms in which BPZE1 affects IL-1*α*, IL-1*β* and IL-33 levels when given prophylactically, but Th2 cytokines (IL-5 and IL-13) when given therapeutically	Not reported	[76]

There is also evidence that BCG, a live-attenuated vaccine based on *Mycobacterium bovis* but designed for use against tuberculosis, protects against heterologous infectious diseases. A phase III double-blinded, placebo-controlled trial carried out late in the COVID-19 pandemic showed that multi-dose BCG protected against COVID-19 disease and indeed all infectious disease (collected as adverse events) [71]. An investigator-blind, randomized, controlled trial conducted in Uganda showed that there were lower incidences of diagnosed non-tuberculosis infectious diseases in those that had received BCG at birth vs those who had delayed BCG administration [72]. Furthermore, *in vitro* growth inhibition assays showed a significant reduction in the growth of *Escherichia coli* and *Klebsiella pneumoniae* following BCG vaccination [73].

### Attenuated pathogens protect against non-infectious diseases

BPZE1 has been shown to protect mice in models of non-infectious disease, including asthma. In an ovalbumin (OVA)-induced model of asthma, BPZE1 protected against airway pathology, decreasing airway inflammation and reducing levels of Th2 cytokines [74]. Virulent *B. pertussis* infection did not protect against the asthmatic phenotypes but exacerbated them instead. BPZE1 was shown to decrease the levels of total cell, eosinophil, macrophage, neutrophil and lymphocyte influx in the lungs, as well as total and OVA-specific serum IgE levels [75]. BPZE1 also reduced levels of Th1 (IL-1*β* and IL-2), Th2 (IL-4, IL-5 and IL-13) cytokines and IL-10 in the bronchial fluid. In the same study, BPZE1 also protected against dinitrochlorobenzene (DNCB)-induced contact hypersensitivity (CHS) in mice, a model for allergic contact dermatitis. When BPZE1 was given intranasally to mice before induction of CHS, there was a reduction in thickness of the ear on DNCB challenge. Two doses of BPZE1, but not one, led to reduced oedema and cellular infiltration compared to non-BPZE1-treated mice, as well as downregulated levels of pro-inflammatory cytokines IL-1*β*, IL-6, IL-2, TNF-*α*, IL-17 and IL-4 in ear homogenates. What is remarkable about the CHS model is that nasal administration of BPZE1 protected against an allergic disease at a site distant from the nose and lungs.

BPZE1 also protected mice in a model of house dust mite (HDM)-induced allergic airway inflammation (AAI), a more relevant model of asthma than the OVA-induced model [76]. In this study, BPZE1 was effective whether given once prophylactically or therapeutically, or twice in a model of severe AAI in which mice were subjected to two rounds of HDM challenge. In all cases, BPZE1 reduced resistance to nebulized methacholine in the lungs, a direct measure of airway resistance and breathing capacity. Mucus production was typically decreased, as well as influx of total cells and eosinophils into the lungs and levels of HDM-specific serum IgG1. In the prophylactic model, levels of IL-1*α*, IL-1*β* and IL-33 were reduced in lung homogenates of BPZE1-treated mice, as were the levels of the chemokine CXCL10. In contrast, in the model of severe AAI, BPZE1 induced a reduction in levels of Th2 cytokines IL-5 and IL-13 in lung homogenates, as well as the chemokines CXCL10, CCL17 and CCL11. Furthermore, two doses of BPZE induced an enhanced reduction for these cytokines.

Vaccination with live-attenuated microbes can reduce overall mortality beyond protection from the disease from which they were designed to protect [77]. This has been observed for measles vaccination in developing countries [78,79] and oral polio vaccination [80,81], and an association between vaccinia scar and survival has been shown [82,83]. BCG was approved in the US for stage I bladder cancer treatment in 1990 [84]. For this indication, BCG is delivered directly into the bladder, where it adheres to the bladder wall and is internalized by tumour cells and urothelial cells. There is a dual mechanism involving both a direct cytotoxic effect and an immune-mediated anti-tumour effect via an inflammatory response recruiting immune cells, including neutrophils, which act to induce apoptosis in tumour cells. Efficacy is limited, with 30–45% of patients failing to respond to treatment [85], although BCG remains one of the most effective therapies for superficial bladder cancer.

There is also evidence suggesting that BCG protects against allergic diseases, possibly by inducing a potent Th1 T-cell response countering the Th2-polarized response in patients with allergic diseases. BCG protected against allergic asthma in multiple mouse models (reviewed in [46], lasting for at least 26 weeks [86], or 46 weeks [87] in some models. Different mechanisms have been suggested, including the induction of a potent Th1 response (and decreasing Th2 cytokines), rebalancing the IL-17/IFN-*γ* ratio [88] or the involvement of Tregs and IL-10 [89]. Dendritic cells (DCs) likely play a major role, as mice injected with BCG-stimulated DCs had reduced pathology in an asthma model [90], a mechanism which appears to involve the induction of CD4^+^CD25^+^FoxP3^+^ regulatory T-cells.

BCG has also been linked with benefits in the context of autoimmune diseases such as multiple sclerosis (MS) and T1D, through various mechanisms [91]. The effects of BCG were studied in a clinical trial of 14 patients with early MS [92]. The authors used magnetic resonance imaging to show that gadolinium-enhanced lesions and new and enlarging lesions significantly dropped after BCG vaccination with no major adverse events. A later study showed reduced disease activity and lower occurrence of a second demyelinating event after a single vaccination with BCG in patients who experienced a first demyelinating event [93]. The mechanism surrounding the beneficial effects of BCG in patients with MS is unknown, but it has been suggested that these may be linked to the role of TNF, of which BCG is a potent inducer [94]. Results of a phase I clinical trial investigating multi-dosing BCG in patients with T1D of more than 10-year disease duration showed death of autoreactive T-cells and induction of Tregs [95]. Patients from this trial were followed for 8 years, and by year 3, blood sugar levels had begun to drop and haemoglobin A1c (HbA1c) levels returned to normal, which persisted for 5 years [96]. The mechanism involved a shift in metabolism from oxidative phosphorylation to aerobic glycolysis, which uses serum sugar for energy. A separate case-control study from Turkey showed that individuals with T1D have fewer BCG scars compared to controls [97]. Protection from T1D was linked to the age of vaccination, as administration of BCG in the first month of life was more protective than later vaccination. Further observational studies carried out in the USA on patients who were undergoing BCG therapy for bladder cancer showed that multi-dose therapy was associated with a lowering of HbA1c in individuals with T1D [98]. There was no association with protection from type 2 diabetes, possibly due to concurrent administration of metformin, a diabetes drug, which inhibits the beneficial effect of BCG on the glycolysis pathway.

Live-attenuated *Salmonella* Typhimurium infection was shown to reduce T1D in NOD mice [57]. Protection was observed when bacteria were administered to mice at any time between 4 and 12 weeks of age but was not observed when killed attenuated *Salmonella* were administered. Splenocytes from attenuated *Salmonella*-infected mice transferred diabetes to recipient NOD-*scid* mice, suggesting that the prevention of T1D by attenuated *Salmonella* was not due to an expansion of regulatory T-cells and that functional diabetogenic T-cells were still present. On the other hand, transfer of purified DCs from mice that had been infected with attenuated *S*. Typhimurium significantly decreased the incidence of diabetes in recipient mice [99]. No surviving bacteria were found in the transferred DCs, nor were the DCs carrying demonstrable *Salmonella* antigens. These DCs altered the trafficking of autoreactive T-cells to the pancreas, many of which remained in a naïve state. Given the long-lasting effects of attenuated *Salmonella* infection on T1D and the relatively short half-life of DCs, it is unlikely that transferred DCs which were demonstrated to prevent T1D would have ever been in contact with the *Salmonella*. This suggests a mechanism which involves long-lasting immune changes within the infected mice after short-term infection that prevents the development of T1D, consistent with the hygiene hypothesis.

## Discussion and future perspectives

It is important to note that the examples of immune priming discussed in this review do not hold true for all pathogens. There is extensive evidence that infection with influenza virus increases host susceptibility to secondary bacterial infection, often with increased morbidity and mortality [100,102]. Nevertheless, here, we review microbes from across the pathogen spectrum (opportunistic pathogens, pathogens and parasites with no commensal aspect to their lifecycle and attenuated pathogens) that protect against subsequent infection by a different pathogen or against non-infectious inflammatory diseases. In humans, evidence for this is difficult to show, due to confounding factors such as variation in microbiome structure and diversity [39,41], different environments [41] and different immune histories. Despite this, there is evidence that a range of pathogens can provide heterologous protection against other diseases.

Opportunistic pathogens and attenuated pathogens may have more in common than it would initially seem. Opportunistic pathogens, during asymptomatic carriage, are effectively fulfilling a role similar to that of live-attenuated vaccines: colonizing the host and promoting immune responses which may be beneficial in the face of future infections with other pathogens (and subsequent disease from the opportunist itself). However, as the host context changes, for example, through illness or injury, carriage of opportunistic pathogens becomes a risk factor and the trade-off between beneficial immune priming and potential for disease of endogenous origin becomes unbalanced. On the other hand, live-attenuated vaccines fulfil a comparable role to that of opportunistic pathogens, often transiently colonizing the host, promoting immune priming, but crucially lacking the virulence potential that opportunistic pathogens retain. Further research into the dynamics of colonization by both types of microbes, opportunists and attenuated pathogens, will provide useful insights into the benefits of microbial immune priming to protect against both infectious and non-infectious diseases.

Mechanisms of heterologous protection, whether caused by infections, including asymptomatic colonization by opportunistic pathogens, or live-attenuated vaccines appear to be varied and context dependent. In addition, there are differences between types of heterologous protection conferred by the same microbe. BPZE1 confers short-lived protection against *S. pneumoniae* linked to a decrease in *S. pneumoniae* colonization and invasion, whilst it confers long-lived protection against influenza without reducing the viral load. In the former model, protection may be a form of non-specific resistance modulated by a common induced innate pathway, perhaps the stimulation of antimicrobial peptides or the induction of an influx of neutrophils. In the second model, protection relies on the tolerogenic effect of BPZE1, avoiding immunopathology that is typical of severe influenza disease. This tolerance is similar to BPZE1-mediated protection against asthma and other non-infectious diseases, as well as *Giardia*-mediated heterologous protection against diarrhoeal diseases and BCG- or *Salmonella*-induced protection against T1D. In the latter, the mice tolerate the production of autoreactive T-cells which are not trafficked to the pancreas due to modulated DCs. The fact that different pathogens can provide heterologous protection against the same disease (for example, *S. aureus*, BCG and attenuated *B. pertussis* providing protection against allergic disease) further supports the concept of non-specific resistance based on commonalities in the immune response.

Individual mechanisms of microbial-induced tolerance may vary and be microbe-dependent such as the strong Th1 response stimulated by BCG, a robust Th2 response caused by *Giardia* infection, or an IL-17-dependent protection caused by *B. pertussis* in protection against RSV. Mechanisms of specific microbe-induced tolerance are still being investigated and appear to be complex. The specific microbial molecules involved in heterologous protection are largely unknown. PT does play a role in protection against influenza by pathogenic *B. pertussis*, although PT itself is unable to elicit protection, suggesting that the presence of other microbial molecules is also necessary [55]. One such mechanism that is likely to be involved in many models of heterologous protection is trained innate immunity, sometimes termed innate immune memory. This concept challenges the view that only adaptive immunity can build immunological memory. Broadly, it is characterized by epigenetic reprogramming, leading to sustained changes in gene expression without permanent changes such as mutation and recombination as for adaptive immunity [103]. Trained innate immunity leads to a faster and greater response against a secondary challenge even with heterologous pathogens, meaning that innate immune memory is non-specific compared to specific adaptive recall responses [104]. BCG has been shown to induce trained innate immunity in human monocytes mediated by epigenetic changes, specifically histone 3 lysine 4 trimethylation through the NOD2 receptor signalling pathway [105]. This allowed for an enhanced cytokine response upon stimulation with unrelated bacterial and fungal pathogens. More recently, BCG was shown to reprogramme haemopoietic stem cells, leading to trained innate immunity [106]. This reprogramming of progenitor cells may be responsible for the long-lasting effects of heterologous protection by BCG. Furthermore, helminth products have been shown to lead to trained innate immunity, specifically enhanced IL-10 and IL-1RA expression in macrophages *in vitro* upon restimulation with the same helminth extracts [107]. These findings were reversed by inhibitors of DNA methylation, suggesting an epigenetic mechanism.

It may be that the route of administration of live-attenuated vaccines affects heterologous responses. BPZE1 is intended to be a mucosal vaccine, delivered to the upper respiratory tract via intranasal administration [58], and as such, heterologous protection has mostly been studied in the context of pathologies that affect the respiratory system such as asthma [74,76] and influenza [65]. However, BPZE1 did protect in a model of DNCB-induced CHS on the ear of mice even though BPZE1 was administered intranasally to mice [75]. This suggests that BPZE1 is able to affect the immune response at distant sites, or at least crosstalk of the mucosal immune response across organ systems. BCG is known to affect progenitor immune cells in the bone marrow, although in this case administration was intravenous [106]. Furthermore, as a therapeutic against bladder cancer, BCG is administered directly into the bladder. In contrast, in clinical studies showing heterologous protection of BCG, the vaccine is often given locally and yet appears to have systemic effects. For example, in the clinical study showing protection against COVID-19, BCG was administered intradermally [71]. In the clinical study showing protection against MS, the vaccine was given intracutaneously [93], again suggesting a systemic effect of a locally administered vaccine.

Much remains to be discovered about heterologous protection by pathogenic and non-pathogenic microbes against other pathogens. A better understanding of the mechanistic basis for heterologous protection will aid in the development of better prevention and treatment approaches for both infectious and non-infectious diseases. In the case of infectious diseases, it will also open up new alternatives to antibiotics, in the form of protective microbes, live-attenuated vaccines and immunotherapeutics. These alternative approaches will open up new challenges, as using live microbes to treat disease carries inherent risks. Protective microbes, for example, may be pathogenic in certain contexts, whilst live-attenuated vaccines themselves present challenges to some at-risk groups, such as immunocompromised individuals and people at the extremes of age.

The human body is colonized by a multitude of microbes with pathogenic potential and challenged with external pathogens daily. As with many aspects of microbiology, context appears to be one of the most important factors in dictating whether pathogen infection will result in disease. By further investigating these different contexts, we will gain valuable new insights into both the potentially beneficial and the harmful aspects of microbial infections.

## Abbreviations

AAI: allergic airway inflammation
BCG: Bacillus Calmette–Guérin
CHS: contact hypersensitivity
CST: community state type
DCs: dendritic cells
DNCB: dinitrochlorobenzene
HbA1c: haemoglobin A1c
HDM: house dust mite
MS: multiple sclerosis
NOD: non-obese diabetic
OVA: ovalbumin
PT: pertussis toxin
RSV: respiratory syncytial virus
T1D: type 1 diabetes
